# Serum biomarkers and their association with disease severity and perinatal outcomes in preeclampsia: a retrospective study

**DOI:** 10.1186/s12884-026-09691-0

**Published:** 2026-07-23

**Authors:** Chong Feng, Meng Hou

**Affiliations:** Department of Obstetrics and Gynecology, Beijing Tongren Hospital, Capital Medical University, Beijing, China

**Keywords:** Birth weight, Blood urea nitrogen, Cystatin C, Preeclampsia, Serum calcium, Uric acid

## Abstract

**Background:**

To investigate the associations of pre-delivery serum blood urea nitrogen (BUN), uric acid (UA), cystatin C (CysC), and serum calcium levels with the occurrence of preeclampsia (PE), disease severity, and birth weight, and to evaluate their discriminatory performance in distinguishing severe preeclampsia (sPE) from PE.

**Methods:**

A total of 328 women with singleton pregnancies who delivered at our hospital between January 2020 and December 2022 and met the inclusion and exclusion criteria were retrospectively enrolled, including 113 women in the normal pregnancy group, 125 in the PE group, and 90 in the sPE group. Maternal demographic characteristics, gestational age at blood sampling, gestational age at delivery, birth weight and related perinatal outcomes, blood pressure measurements, and laboratory parameters were collected. Multivariable binary logistic regression analyses were performed to evaluate the associations between the major laboratory biomarkers and the occurrence of PE and sPE after adjustment for maternal age, body mass index (BMI), nulliparity, assisted reproductive technology (ART) conception, and gestational age at blood sampling. Spearman correlation analysis and multiple linear regression analysis were used to assess the associations between the major laboratory biomarkers and birth weight after further adjustment for gestational age at delivery. Receiver operating characteristic (ROC) curve analysis was performed to evaluate the discriminatory performance of the major laboratory biomarkers and the combined model consisting of BUN, UA, and CysC in distinguishing sPE from PE.

The calibration and clinical utility of the combined model were further evaluated using the Hosmer–Lemeshow goodness-of-fit test, calibration plots, and decision curve analysis (DCA).

**Results:**

Significant differences in BUN, UA, CysC, and serum calcium levels were observed among the three groups. After adjustment for maternal age, BMI, nulliparity, ART conception, and gestational age at blood sampling, elevated BUN, UA, and CysC levels were independently associated with the occurrence of PE, with odds ratios (ORs) of 3.057, 4.432, and 6.738, respectively (all *P* < 0.001). Higher serum calcium levels were associated with a lower risk of PE (OR = 0.733, *P* = 0.019).

Among women with PE, only UA remained significantly associated with sPE after adjustment for the above confounding factors (OR = 1.444, 95% confidence interval [CI]: 1.056–1.975, *P* = 0.021). Correlation analyses showed that BUN, UA, and CysC were negatively correlated with birth weight, whereas serum calcium was not significantly associated with birth weight. After further adjustment for gestational age at delivery, only BUN and UA remained significantly associated with reduced birth weight. ROC analysis showed that the area under the curve (AUC) values for BUN, UA, CysC, and serum calcium (evaluated in the lower-value direction) in distinguishing sPE from PE were 0.610, 0.626, 0.567, and 0.590, respectively. The combined model including BUN, UA, and CysC yielded an AUC of 0.627, with a sensitivity of 37.8%, specificity of 87.2%, positive predictive value of 68.0%, and negative predictive value of 66.1%. The Hosmer–Lemeshow goodness-of-fit test indicated no significant lack of model fit, and decision curve analysis demonstrated that the combined model provided greater net benefit than the treat-all and treat-none strategies across a threshold probability range of approximately 0.34–0.66.

**Conclusions:**

Pre-delivery levels of BUN, UA, CysC, and serum calcium were associated with the occurrence of PE, with UA being independently associated with sPE. BUN and UA remained associated with reduced birth weight after adjustment for gestational age at delivery. However, the discriminatory performance of UA, the other individual biomarkers, and the combined model for distinguishing sPE from PE was limited, and they should not be used alone to determine disease severity. Markedly elevated UA levels may help identify women with PE who require closer clinical monitoring; however, disease severity should still be comprehensively assessed based on blood pressure, proteinuria, clinical symptoms, and evidence of organ dysfunction.

## Background

Preeclampsia (PE) is a major subtype of hypertensive disorders of pregnancy and one of the leading obstetric complications associated with adverse maternal and perinatal outcomes [1, 2]. It can affect multiple organ systems, including the kidneys, liver, coagulation system, nervous system, and placental function, and is associated with adverse outcomes such as preterm birth, fetal growth restriction, low birth weight, low Apgar scores, and neonatal intensive care unit admission [2, 3]. Given the substantial heterogeneity in disease progression and maternal–fetal risks among women with PE, accurate assessment of disease severity and risk stratification is of great importance for clinical surveillance, treatment decision-making, and determination of the optimal timing of delivery [2].

Placental hypoperfusion, endothelial dysfunction, and systemic inflammatory responses are key pathophysiological mechanisms involved in the development and progression of PE [4]. The kidney is one of the earliest target organs affected in PE.

Glomerular endothelial injury and alterations in renal hemodynamics may lead to reduced glomerular filtration function and abnormalities in various renal function-related biomarkers [5]. Blood urea nitrogen (BUN), uric acid (UA), and cystatin C (CysC) are all closely associated with renal function. Elevated UA levels are commonly observed in women with PE and may be related to oxidative stress, endothelial dysfunction, and inflammatory responses [6]. CysC has also been used as a marker of changes in glomerular filtration function. Therefore, these biomarkers may reflect PE-related renal involvement and disease severity from different perspectives.

In addition to renal function-related biomarkers, alterations in calcium metabolism and coagulation function may also be associated with disease progression in PE. 

Calcium ions play an important role in the regulation of vascular tone, and changes in serum calcium levels may reflect metabolic and vascular functional status in some patients.

Previous systematic reviews have shown that circulating calcium levels are lower in women with PE than in those with normal pregnancies [7]. Furthermore, PE is characterized by endothelial injury and alterations in the coagulation–fibrinolytic system, and severe cases may develop coagulation abnormalities. A recent systematic review also suggested that fibrinogen and D-dimer levels are altered in women with PE [8]. Therefore, in addition to evaluating major biomarkers such as BUN, UA, CysC, and serum calcium, descriptive comparisons of coagulation-related parameters may provide a more comprehensive understanding of the clinical characteristics of PE across different severity levels.

Previous studies have suggested that UA, CysC, and serum calcium levels are associated with PE and related adverse pregnancy outcomes [7, 9]. However, the roles of these routinely available laboratory biomarkers in stratifying PE severity remain incompletely understood, and some studies have not adequately accounted for potential confounding factors such as gestational age at blood sampling, maternal body mass index (BMI), and parity. In addition, reduced birth weight is commonly observed in women with severe preeclampsia (sPE), whereas the impact of PE on birth weight may vary according to gestational age. Therefore, gestational age at delivery should be considered when evaluating the association between laboratory biomarkers and birth weight [10].

Accordingly, the present retrospective study analyzed data from women with singleton pregnancies who delivered at our hospital. We compared major laboratory biomarkers among normal pregnancy, PE, and sPE groups and further evaluated their associations with PE severity and birth weight, with the aim of providing additional evidence for clinical severity assessment and risk stratification in women with PE.

## Methods

### Study design and population

This was a single-center retrospective study, and the manuscript was reported in accordance with the Strengthening the Reporting of Observational Studies in Epidemiology (STROBE) statement. Clinical data were retrospectively collected from 328 women with singleton pregnancies who delivered at our hospital between January 2020 and December 2022 and met the inclusion and exclusion criteria. Among them, 113 women were included in the normal pregnancy group, 125 in the PE group, and 90 in the sPE group.

The diagnoses of PE and sPE were established according to national and international guidelines for hypertensive disorders of pregnancy, in conjunction with the recommendations of the American College of Obstetricians and Gynecologists (ACOG) and the International Society for the Study of Hypertension in Pregnancy (ISSHP) [2, 11]. The PE group included women who met the diagnostic criteria for PE but did not fulfill the criteria for sPE. The sPE group included women who met the diagnostic criteria for PE and presented with severe clinical features. The normal pregnancy group consisted of women with singleton pregnancies who delivered during the same period and had no hypertensive disorders of pregnancy or other major pregnancy-related complications.

The inclusion criteria were as follows: singleton pregnancy; delivery at our hospital between January 2020 and December 2022; and complete clinical and major laboratory data. The exclusion criteria were: multiple pregnancy; pre-existing conditions that might affect the interpretation of major laboratory biomarkers, including chronic hypertension, pregestational diabetes mellitus, chronic liver or kidney disease, established heart disease, and autoimmune diseases; severe fetal structural abnormalities; intrauterine fetal demise; or substantial missing clinical data.

This study was approved by the Ethics Committee of Beijing Tongren Hospital, Capital Medical University (approval number: TREC2025-KY107). As a retrospective study, all data were obtained from existing medical records and anonymized prior to statistical analysis. The requirement for informed consent was handled in accordance with the approval and regulations of the ethics committee.

### Outcome measures

Clinical data were retrospectively collected from the hospital electronic medical record system. Maternal characteristics included age, body mass index (BMI), nulliparity status, and conception by assisted reproductive technology (ART).

Perinatal data included gestational age at blood sampling, gestational age at delivery, neonatal birth weight, 1-minute and 5-minute Apgar scores, and neonatal intensive care unit (NICU) admission. Blood pressure data included systolic blood pressure and diastolic blood pressure.

Laboratory data were obtained from the hospital electronic medical record and laboratory information systems. The last available biochemical and coagulation test results before delivery were extracted for analysis, and the corresponding gestational age at blood sampling was recorded. The last laboratory measurements before delivery were selected because they were obtained closest to the time of clinical decision-making regarding delivery and were therefore considered to best reflect the patients’ laboratory status immediately before delivery.

The primary laboratory biomarkers included BUN, UA, CysC, and serum calcium. Coagulation-related parameters included activated partial thromboplastin time (APTT), fibrinogen, and D-dimer.

Serum biochemical parameters were measured using an AU5821 fully automated biochemical analyzer (Beckman Coulter, USA), while coagulation parameters were measured using a SYSMEX CS-5100 fully automated coagulation analyzer (Sysmex Corporation, Japan). All laboratory tests were performed by the hospital clinical laboratory according to standard operating procedures (SOPs).

Because gestational age at blood sampling varied among participants, gestational age at blood sampling was included as a potential confounding factor and adjusted for in subsequent multivariable analyses.

### Statistical analysis

Statistical analyses were performed using SPSS version 25.0 (IBM Corp., Armonk, NY, USA). Continuous variables were first tested for normality. Variables with a normal distribution were presented as mean ± standard deviation (SD), and comparisons among the three groups were performed using one-way analysis of variance (ANOVA). Welch’s correction was applied when the assumption of homogeneity of variance was violated. Variables with a non-normal distribution were presented as median and interquartile range [M (P25, P75)] and compared using the Kruskal–Wallis H test. Categorical variables were expressed as numbers and percentages [n (%)] and compared using the chi-square test or Fisher’s exact test, as appropriate.

Multivariable binary logistic regression analyses were performed to evaluate the associations of major laboratory biomarkers with the occurrence of PE and the presence of sPE. Separate regression models were constructed to compare the PE cases with the normal pregnancy group and the sPE group with the PE group. 

Standardized BUN, UA, CysC, and serum calcium values were entered into the models individually, with adjustment for maternal age, BMI, nulliparity, ART conception, and gestational age at blood sampling. Results were reported as odds ratios (ORs) with 95% confidence intervals (CIs).

To assess the robustness of the primary findings with respect to variations in gestational age at blood sampling, a sensitivity analysis was further performed by restricting the study population to women whose blood samples were collected between 36 + 0 and 38 + 6 weeks of gestation, and the multivariable logistic regression analyses were repeated.

Spearman correlation analysis was used to evaluate the relationships between major laboratory biomarkers and neonatal birth weight. Multiple linear regression analysis was subsequently performed with adjustment for gestational age at delivery.

Receiver operating characteristic (ROC) curve analysis was performed to assess the discriminatory ability of the major laboratory biomarkers and the combined model in distinguishing sPE from PE. The area under the curve (AUC) and its 95% CI were calculated. Optimal cutoff values were determined using the Youden index. Sensitivity, specificity, positive predictive value (PPV), and negative predictive value (NPV) were also calculated. For the combined biomarker model, predicted probabilities derived from logistic regression analysis were used for ROC analysis. Model calibration was further evaluated using the Hosmer–Lemeshow goodness-of-fit test and calibration plots, and clinical utility was assessed using decision curve analysis (DCA).

All statistical tests were two-sided, and a P value < 0.05 was considered statistically significant.

## Results

### Comparison of maternal characteristics, perinatal outcomes, and laboratory biomarkers among the three groups

**Fig. 1:**
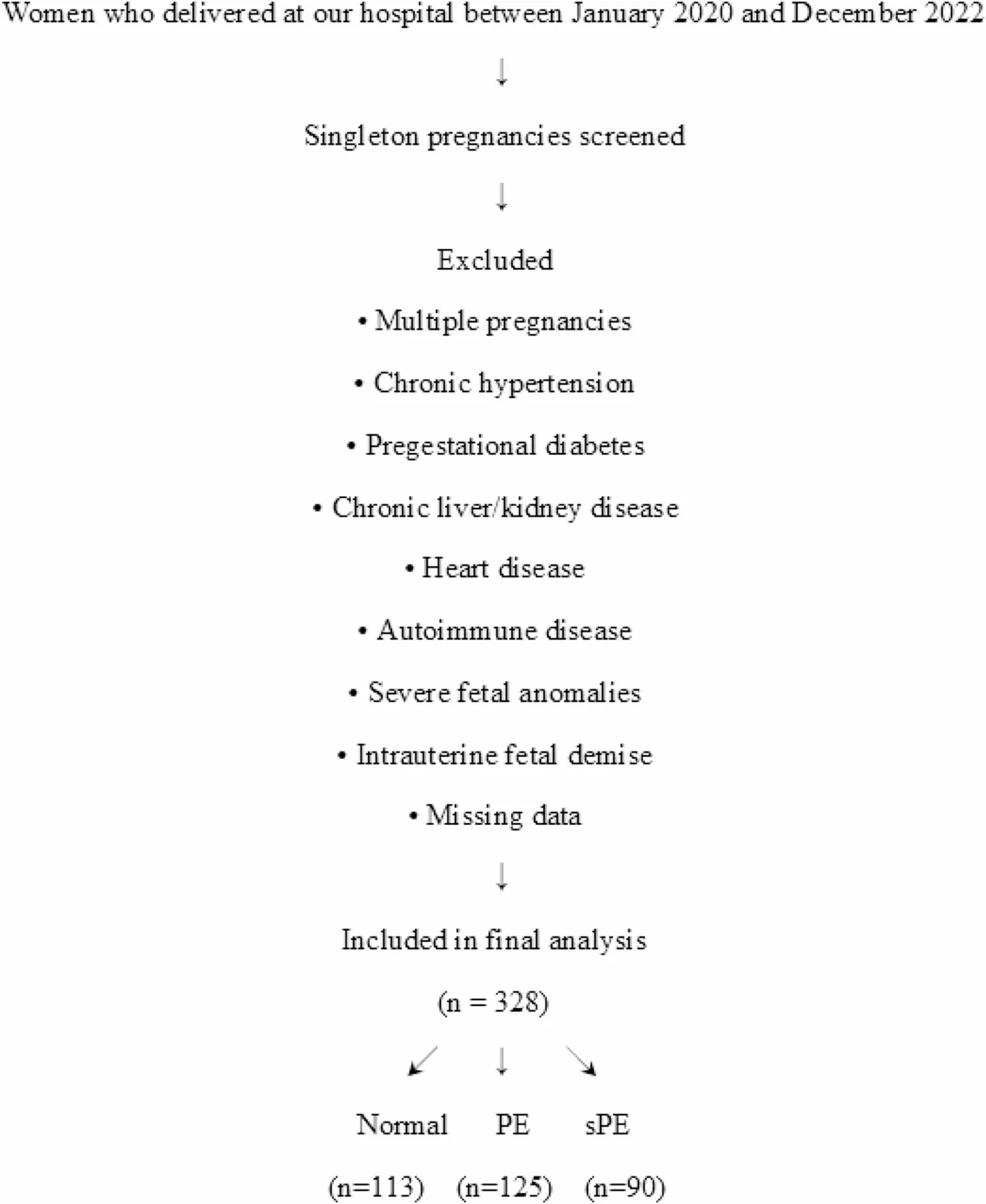
Flow diagram of participant selection

A total of 328 women with singleton pregnancies were included in this study, comprising 113 women in the normal pregnancy group, 125 in the PE group, and 90 in the sPE group. The participant selection process is shown in Fig. 1. The median (interquartile range) gestational age at blood sampling was 36.29 (36.00–36.64) weeks, 38.00 (36.57–39.14) weeks, and 36.86 (34.54–38.47) weeks in the normal pregnancy, PE, and sPE groups, respectively, with a statistically significant difference among the three groups (*P* < 0.001). Therefore, gestational age at blood sampling was included as a potential confounding factor in the subsequent multivariable analyses.

Significant differences were observed among the three groups in maternal age, BMI, proportion of nulliparous women, gestational age at blood sampling, gestational age at delivery, neonatal birth weight, 1-minute and 5-minute Apgar scores, NICU admission rate, systolic blood pressure, and diastolic blood pressure (all *P* < 0.05). No significant difference was found in the proportion of pregnancies conceived by ART (*P* = 0.586).

Significant differences were also observed among the three groups in BUN, UA, CysC, serum calcium, and fibrinogen levels (all *P* < 0.05), whereas APTT and D-dimer levels did not differ significantly among the groups (all *P* > 0.05). Compared with the normal pregnancy group, women in both the PE and sPE groups had higher levels of BUN, UA, and CysC, whereas women in the sPE group had relatively lower serum calcium levels. Detailed comparisons of maternal characteristics, perinatal outcomes, and laboratory biomarkers are presented in Table 1.

**Table 1:** Comparison of maternal characteristics, perinatal outcomes, and laboratory biomarkers among the normal pregnancy, PE, and sPE groups

Variable	Normal pregnancy group (*n* = 113)	PE group (*n* = 125)	sPE group (*n* = 90)	*P* value
Maternal age, years	31.44 ± 3.47	31.15 ± 4.56	32.87 ± 4.03	0.008
BMI, kg/m²	21.63 (19.98, 23.27)	23.03 (20.70, 25.55)	22.83 (20.31, 25.56)	0.001
Nulliparous, n (%)	69 (61.1)	104 (83.2)	69 (76.7)	< 0.001
ART conception, n (%)	4 (3.5)	7 (5.6)	6 (6.7)	0.586
Gestational age at blood sampling, weeks	36.29 (36.00, 36.64)	38.00 (36.57, 39.14)	36.86 (34.54, 38.47)	< 0.001
Gestational age at delivery, weeks	39.14 (38.71, 39.86)	38.57 (37.64, 39.57)	37.50 (34.71, 38.71)	< 0.001
Birth weight, g	3320.00 ± 323.60	3079.28 ± 428.20	2680.56 ± 741.82	< 0.001
1-min Apgar score	10.00 (10.00, 10.00)	10.00 (10.00, 10.00)	10.00 (9.00, 10.00)	< 0.001
5-min Apgar score	10.00 (10.00, 10.00)	10.00 (10.00, 10.00)	10.00 (10.00, 10.00)	0.006
NICU admission, n (%)	8 (7.1)	23 (18.4)	45 (50.0)	< 0.001
BUN, mmol/L	2.90 (2.40, 3.45)	3.50 (2.95, 4.35)	4.15 (2.80, 5.30)	< 0.001
UA, µmol/L	290.68 ± 60.85	371.41 ± 85.59	412.94 ± 99.12	< 0.001
CysC, mg/L	0.93 (0.81, 1.05)	1.18 (1.03, 1.35)	1.23 (1.05, 1.48)	< 0.001
Serum calcium, mmol/L	2.23 ± 0.08	2.24 ± 0.12	2.18 ± 0.17	0.020
APTT, s	26.10 (24.90, 27.30)	26.20 (25.05, 27.85)	26.20 (24.60, 27.50)	0.473
Fibrinogen, g/L	4.30 (3.95, 4.68)	4.53 (3.91, 5.06)	3.92 (3.41, 4.52)	< 0.001
D-dimer, mg/L	1.58 (1.16, 2.23)	1.80 (1.17, 2.69)	1.62 (1.11, 2.29)	0.201
Systolic blood pressure, mmHg	116.00 (110.00, 123.00)	144.00 (140.00, 149.00)	160.00 (153.00, 170.00)	< 0.001
Diastolic blood pressure, mmHg	75.00 (70.00, 80.00)	94.00 (91.00, 97.00)	100.00 (93.75, 107.00)	< 0.001

Continuous variables are presented as mean±standard deviation (SD) or median (P25, P75), and categorical variables are presented as number (percentage)

*P* values represent overall comparisons among the three groups

### Multivariable logistic regression analysis of major laboratory biomarkers associated with the occurrence of PE

Using the normal pregnancy group as the reference group, women with PE and sPE were combined into a single case group for multivariable binary logistic regression analysis. After adjustment for maternal age, BMI, nulliparity, ART conception, and gestational age at blood sampling, higher standardized BUN, UA, and CysC levels were independently associated with the occurrence of PE, whereas higher standardized serum calcium levels were associated with a lower risk of PE. The results are shown in Table 2.

**Table 2:** Multivariable logistic regression analysis of major laboratory biomarkers associated with the occurrence of PE

Variable	OR	95% CI	*P* value
Standardized BUN	3.057	2.112–4.426	< 0.001
Standardized UA	4.432	2.975–6.601	< 0.001
Standardized CysC	6.738	4.008–11.327	< 0.001
Standardized serum calcium	0.733	0.566–0.951	0.019

All major laboratory biomarkers were standardized before being entered into the regression model

*OR* Odds ratio, *CI* Confidence interval

### Multivariable logistic regression analysis of major laboratory biomarkers associated with sPE

A multivariable logistic regression analysis was performed among women with PE. After adjustment for maternal age, BMI, nulliparity, ART conception, and gestational age at blood sampling, only elevated standardized UA remained significantly associated with sPE. The associations of standardized BUN, CysC, and serum calcium with sPE did not reach statistical significance. The results are shown in Table 3.

**Table 3:** Multivariable logistic regression analysis of major laboratory biomarkers associated with sPE

Variable	OR	95% CI	*P* value
Standardized BUN	1.316	0.983–1.762	0.065
Standardized UA	1.444	1.056–1.975	0.021
Standardized CysC	1.211	0.900–1.629	0.206
Standardized serum calcium	0.781	0.595–1.025	0.075

All major laboratory biomarkers were standardized before being entered into the regression model

*OR* Odds ratio, *CI* Confidence interval

To evaluate the robustness of the primary findings with respect to variations in gestational age at blood sampling, a sensitivity analysis was performed by restricting the study population to women whose blood samples were collected between 36 + 0 and 38 + 6 weeks of gestation. A total of 199 women were included, comprising 79 women in the normal pregnancy group, 77 in the PE group, and 43 in the sPE group.

After adjustment for maternal age, BMI, nulliparity, ART conception, and gestational age at blood sampling, elevated standardized BUN, UA, and CysC levels remained independently associated with the occurrence of PE, with ORs of 2.802 (95% CI: 1.525–5.147, *P* = 0.001), 3.483 (95% CI: 2.046–5.930, *P* < 0.001), and 6.281 (95% CI: 3.009–13.110, *P* < 0.001), respectively. The association between serum calcium and PE was no longer statistically significant (OR = 1.258, 95% CI: 0.803–1.971, *P* = 0.316).

Among women with PE, the direction of the association between UA and sPE remained consistent with that observed in the primary analysis; however, the association was of borderline statistical significance (OR = 1.536, 95% CI: 0.997–2.365, *P* = 0.052). BUN, CysC, and serum calcium were not significantly associated with sPE.

Overall, the sensitivity analysis supported the robustness of the primary findings.

However, the subgroup analysis for sPE should be interpreted with caution because of the reduced sample size and the consequent reduction in statistical power.

### Associations between major laboratory biomarkers and birth weight

Spearman correlation analysis showed that BUN, UA, and CysC were negatively correlated with neonatal birth weight, whereas serum calcium was not significantly associated with birth weight. After further adjustment for gestational age at delivery, standardized BUN and UA remained significantly associated with reduced birth weight, whereas the associations of standardized CysC and serum calcium with birth weight did not reach statistical significance. The results are shown in Table 4.

**Table 4:** Associations between major laboratory biomarkers and birth weight

Variable	Spearman correlation coefficient (rs)	*P* value	B coefficient (adjustment for gestational age at delivery)	95% CI	Standardized β	*P* value
Standardized BUN	-0.389	< 0.001	-116.074	-157.365 to -74.783	-0.206	< 0.001
Standardized UA	-0.418	< 0.001	-102.792	-144.406 to -61.178	-0.182	< 0.001
Standardized CysC	-0.264	< 0.001	-34.711	-76.013 to 6.591	-0.062	0.099
Standardized serum calcium	0.095	0.087	27.159	-13.180 to 67.499	0.048	0.186

B coefficients, 95% confidence intervals (CIs), and standardized β values were derived from multiple linear regression analyses adjusted for gestational age at delivery

*CI* Confidence interval

### ROC analysis of major laboratory biomarkers for discriminating sPE

ROC analysis showed that the AUC values of BUN, UA, CysC, and serum calcium (evaluated in the lower-value direction) for discriminating sPE from preeclampsia PE were all below 0.70, indicating limited discriminatory ability for each individual biomarker. The combined model including BUN, UA, and CysC yielded an AUC of 0.627, which was slightly higher than those of the individual biomarkers; however, its overall discriminatory performance remained limited.

The Hosmer–Lemeshow goodness-of-fit test showed no significant difference between the predicted probabilities and the observed outcomes (χ² = 11.460, df = 8, *P* = 0.177), indicating no evidence of poor model fit. The calibration plot demonstrated good overall agreement between the predicted and observed probabilities, although some deviations were observed. These findings suggest that the combined model showed reasonable calibration but remained limited in its ability to discriminate sPE from PE.

**Fig. 2:**
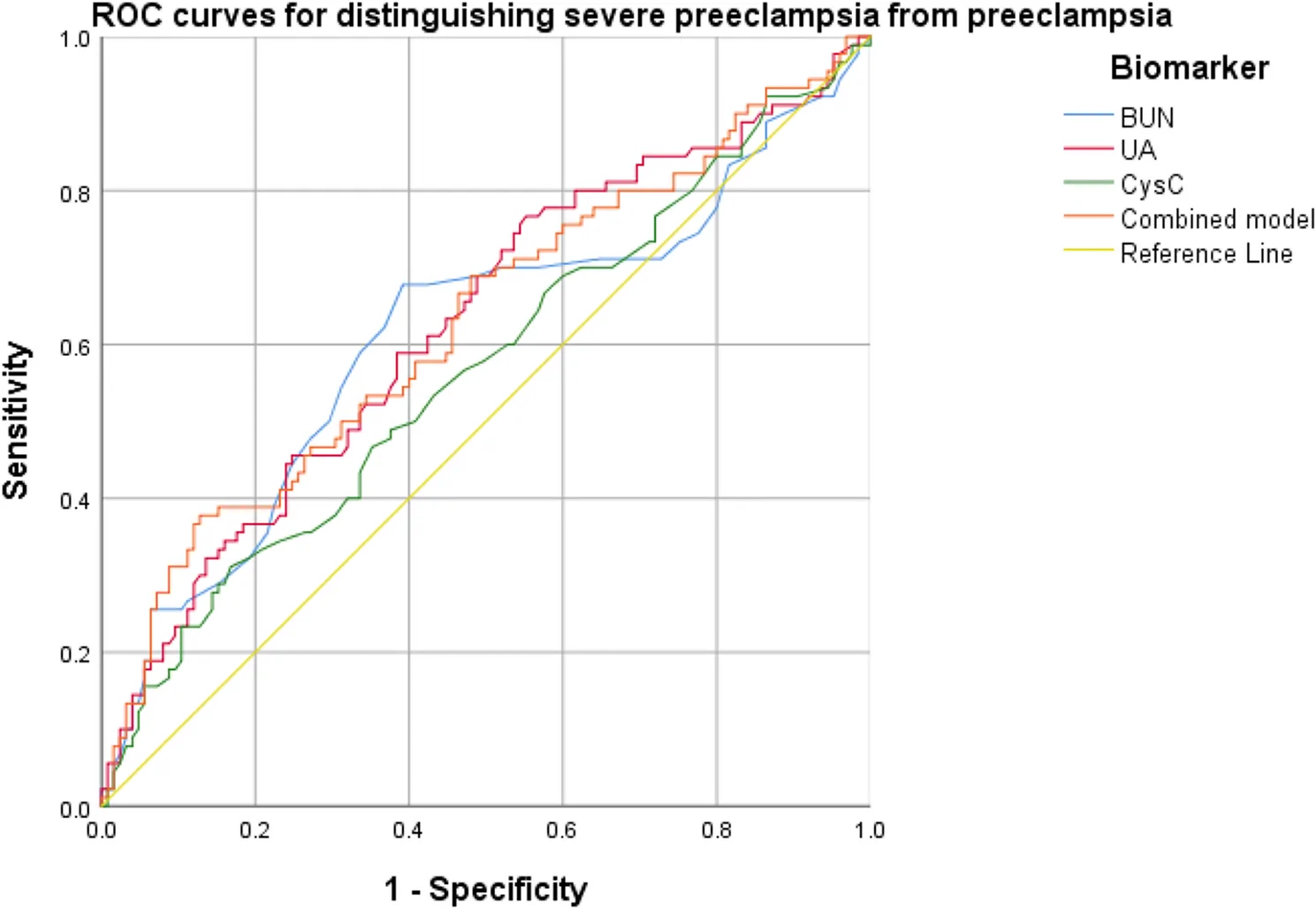
ROC curves of BUN, UA, CysC, and the combined model for discriminating sPE. Note: The combined model included BUN, UA, and CysC. ROC, Receiver operating characteristic curve

**Fig. 3:**
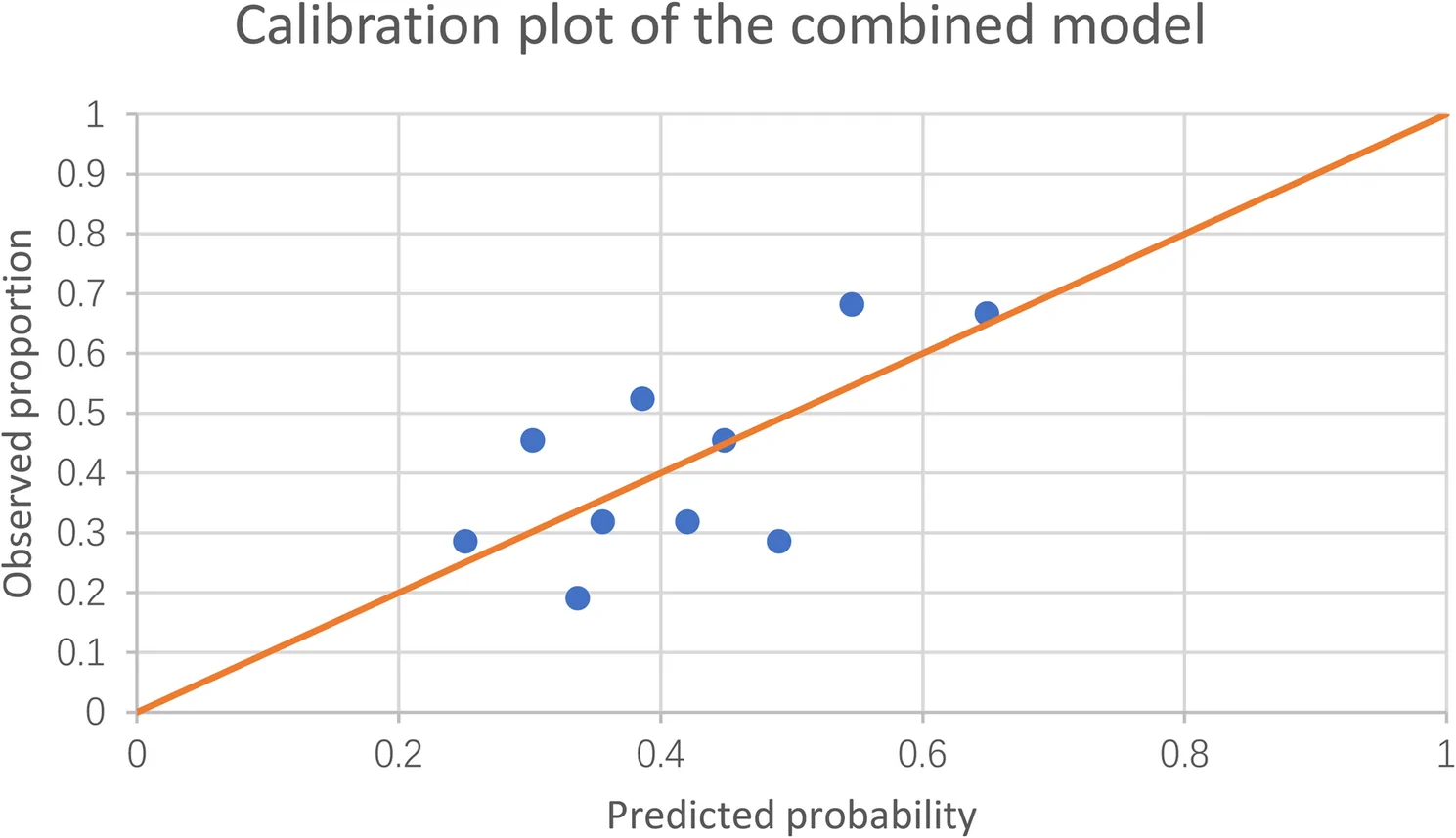
Calibration plot of the combined model including BUN, UA and CysC for discriminating sPE from PE

**Fig. 4:**
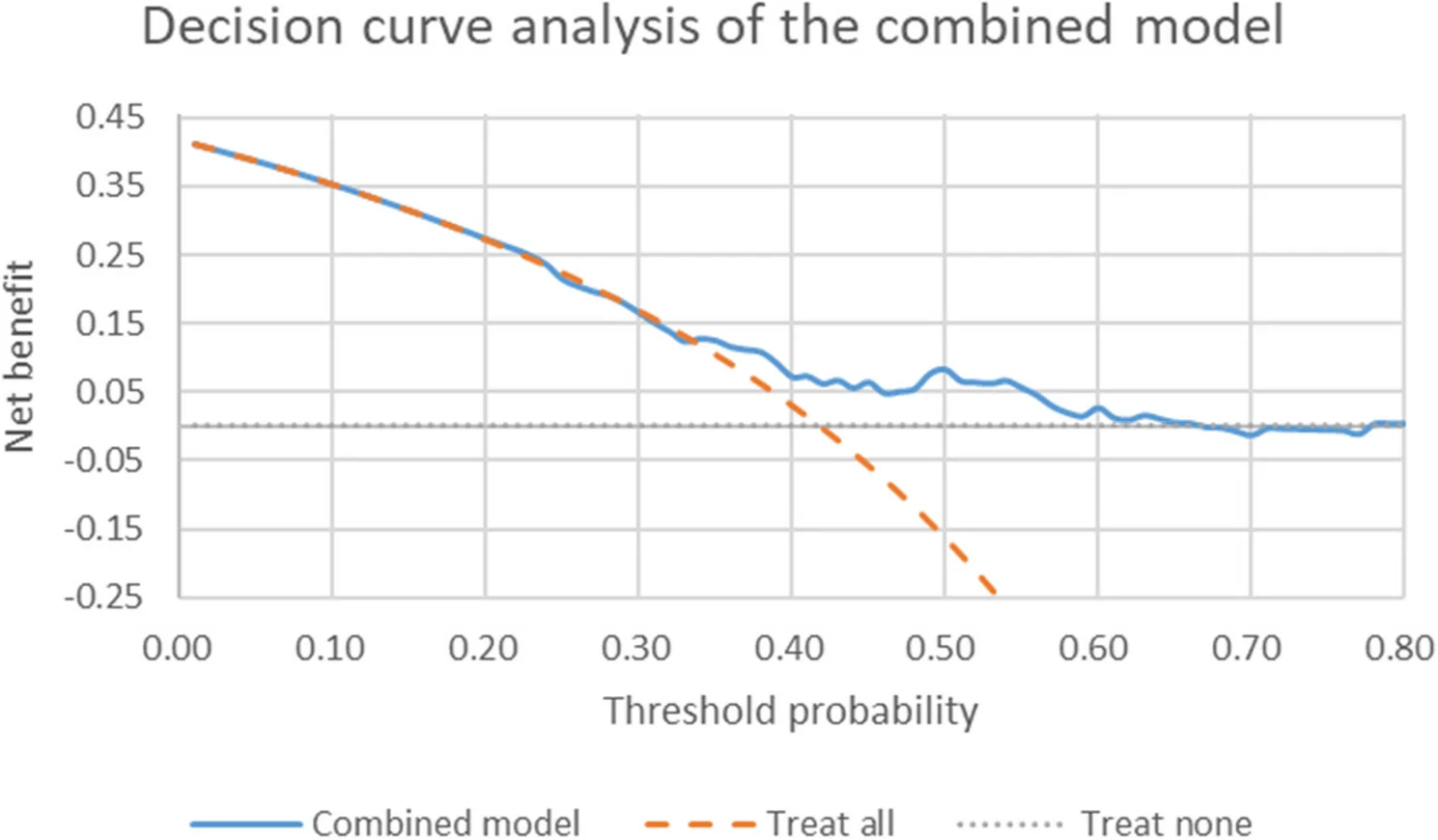
DCA of the combined model including BUN, UA and CysC for discriminating sPE from PE

DCA demonstrated that the combined model provided greater net benefit than the treat-all and treat-none strategies across a threshold probability range of approximately 0.34–0.66, suggesting potential clinical utility within this threshold probability range. However, considering that the AUC of the combined model remained below 0.70, its clinical applicability should be interpreted with caution, and it should not be used alone to determine the severity of preeclampsia. The results are presented in Table 5; Figs. 2, 3 and 4.

**Table 5:** ROC analysis of major laboratory biomarkers for discriminating sPE

Variable	AUC	95% CI	*P* value	Optimal cutoff value	Sensitivity	Specificity	PPV	NPV
BUN	0.610	0.531–0.690	0.006	3.75 mmol/L	67.8%	60.8%	55.5%	72.4%
UA	0.626	0.550–0.702	0.002	348.5 µmol/L	76.7%	44.8%	50.1%	72.7%
CysC	0.567	0.489–0.646	0.093	1.405 mg/L	31.1%	83.2%	57.1%	62.6%
Serum calcium (lower-value direction)	0.590	0.510–0.670	0.025	≤ 2.145 mmol/L	43.3%	80.0%	60.9%	66.2%
Combined model	0.627	0.550–0.704	0.001	0.4966	37.8%	87.2%	68.0%	66.1%

Serum calcium was analyzed in the lower-value direction. The combined model included BUN, UA, and CysC, and the cutoff value was based on the predicted probability generated by logistic regression analysis

*AUC* Area under the curve, *CI* Confidence interval, *PPV* Positive predictive value, *NPV* Negative predictive value

## Discussion

Based on data from 328 women with singleton pregnancies, this study compared major laboratory biomarkers among normal pregnancy, PE, and sPE groups and further evaluated their associations with the occurrence of PE, severe disease, and birth weight. The results showed that BUN, UA, and CysC levels were elevated in women with PE, whereas serum calcium levels were relatively lower in women with sPE. Multivariable logistic regression analysis demonstrated that, after adjustment for maternal age, BMI, nulliparity, ART conception, and gestational age at blood sampling, BUN, UA, CysC, and serum calcium were all associated with the occurrence of PE. Among women with PE, however, only UA remained significantly associated with sPE. After further adjustment for gestational age at delivery, BUN and UA remained associated with reduced birth weight. ROC analysis indicated that BUN, UA, CysC, and the combined model had limited ability to discriminate sPE from PE.

Renal involvement is common in women with PE, and glomerular endothelial injury and alterations in renal hemodynamics may lead to impaired renal function [5]. In the present study, BUN, UA, and CysC levels were all higher in women with PE than in those with normal pregnancies, and these associations remained significant after adjustment for maternal age, BMI, nulliparity, ART conception, and gestational age at blood sampling. These findings are generally consistent with previous studies reporting elevated urea and UA levels in women with PE [12], and are also in agreement with studies showing increased CysC levels in PE and suggesting that CysC may reflect PE-related renal impairment [13, 14]. Among these biomarkers, UA not only reflects renal excretory function but may also be associated with oxidative stress, endothelial dysfunction, inflammatory responses, and placental abnormalities, which has led to considerable interest in its role in the pathogenesis and progression of PE [6]. Furthermore, our study found that, among women with PE, only UA remained significantly associated with sPE after adjustment for relevant confounding factors. Previous meta-analyses have similarly reported higher serum UA levels in women with severe PE, eclampsia, and HELLP syndrome and suggested associations with disease severity and pregnancy complications [15]. Therefore, compared with BUN and CysC, UA may better reflect disease progression in PE.

Changes in serum calcium levels were also of particular interest in this study. We found that serum calcium levels were lower in the sPE group than in both the normal pregnancy and PE groups, and that higher standardized serum calcium levels were associated with a lower risk of PE in the multivariable logistic regression model.

Previous systematic reviews have shown that circulating calcium levels are lower in women with PE than in those with normal pregnancies, suggesting that abnormalities in calcium metabolism may be involved in the development and progression of PE [7].

However, when further distinguishing sPE from PE, serum calcium was not significantly associated with sPE, and ROC analysis demonstrated limited discriminatory ability. These findings suggest that serum calcium may primarily reflect PE-related metabolic and vascular functional changes and may not be suitable as an independent marker of disease severity.

This study also evaluated the associations between major laboratory biomarkers and neonatal birth weight. Spearman correlation analysis demonstrated that BUN, UA, and CysC were negatively correlated with birth weight. However, after adjustment for gestational age at delivery, only BUN and UA remained significantly associated with reduced birth weight, whereas the association between CysC and birth weight was no longer statistically significant. Previous studies have reported associations between maternal UA and CysC levels in late pregnancy and adverse birth outcomes [9]. In addition, the impact of PE on birth weight may vary according to gestational age; therefore, gestational age at delivery should be considered when evaluating the relationship between laboratory biomarkers and birth weight [10]. Our findings indicate that the associations of BUN and UA with reduced birth weight persisted after adjustment for gestational age at delivery, suggesting that these biomarkers may be related to placental hypoperfusion, disease severity, and the risk of fetal growth restriction. Nevertheless, further studies are needed to confirm these observations.

ROC analysis showed that the AUC values of BUN, UA, CysC, and serum calcium (evaluated in the lower-value direction) for discriminating sPE from PE were all below 0.70. Although the combined model including BUN, UA, and CysC achieved a slightly higher AUC than the individual biomarkers, its overall discriminatory performance remained limited. In the present study, the optimal cutoff value of UA for discriminating sPE from PE was 348.5 µmol/L, suggesting that women with PE and markedly elevated UA levels may require closer clinical surveillance. However, UA levels and their optimal cutoff values may vary across studies because of differences in study populations, gestational age, disease definitions, laboratory methods, and sample size. Moreover, the cutoff identified in the present study was derived from a single-center retrospective dataset, and the AUC of UA was only 0.626. Therefore, external validation in independent cohorts is required before this cutoff can be considered for clinical application, and it should not be used as an independent criterion for identifying sPE. Previous systematic reviews have also suggested that current evidence is insufficient to support the use of UA alone for predicting adverse maternal and perinatal outcomes [16]. Therefore, these routinely available laboratory biomarkers are more appropriately used as supplementary tools for clinical risk assessment rather than as standalone indicators of disease severity. Assessment of disease severity should continue to rely on a comprehensive evaluation of blood pressure, proteinuria, clinical symptoms, and evidence of organ dysfunction [2]. The present study further evaluated the potential clinical utility of the combined model using DCA. The combined model provided greater net benefit than the treat-all and treat-none strategies across a threshold probability range of approximately 0.34–0.66, suggesting potential clinical value within this range. Nevertheless, because the AUC of the combined model remained below 0.70 and no external validation was performed, the model should not be used directly for clinical decision-making regarding disease severity stratification.

Several limitations of this study should be acknowledged. First, this was a single-center retrospective study with a relatively limited sample size, and the findings may have been influenced by selection bias and the completeness of available clinical data. Therefore, validation in larger multicenter studies is warranted. Second, the analysis was based on the last laboratory assessment before delivery.

Although gestational age at blood sampling was adjusted for in the multivariable models, the potential influence of differences in sampling time could not be completely eliminated. Third, this study was based on routinely collected clinical laboratory data. Although all laboratory tests were performed by the hospital clinical laboratory according to standard operating procedures and quality-control requirements, the retrospective nature of the study precluded further verification of specific reagent batches and assay performance parameters. Fourth, the optimal cutoff values identified by ROC analysis and the combined biomarker model were both derived from this single-center dataset and have not been externally validated.

Although additional calibration analysis and DCA were performed to further evaluate the model, these findings should not be directly generalized to clinical decision-making without external validation. Fifth, as an observational study, our findings demonstrate associations rather than causal relationships.

## Conclusions

Pre-delivery BUN, UA, CysC, and serum calcium levels were associated with the occurrence of PE, and UA was independently associated with severe PE. BUN and UA remained associated with reduced birth weight after adjustment for gestational age at delivery. However, ROC analysis showed that the AUC of UA for discriminating severe PE was only 0.626, and the discriminatory performance of BUN, CysC, and the combined model was also limited. Therefore, these routinely available laboratory biomarkers should not be used alone to determine disease severity. Women with PE and markedly elevated UA levels may require closer clinical surveillance; however, assessment of disease severity should continue to rely on a comprehensive evaluation of blood pressure, proteinuria, clinical symptoms, and evidence of organ dysfunction.

## Data Availability

The datasets used and/or analysed during the current study are available from the corresponding author on reasonable request.

## Abbreviations

PE: Preeclampsia
sPE: Severe preeclampsia
BUN: Blood urea nitrogen
UA: Uric acid
CysC: Cystatin C
BMI: Body mass index
ART: Assisted reproductive technology
NICU: Neonatal intensive care unit
APTT: Activated partial thromboplastin time
SD: Standard deviation
OR: Odds ratio
CI: Confidence interval
ROC: Receiver operating characteristic
AUC: Area under the curve
DCA: Decision curve analysis
PPV: Positive predictive value
NPV: Negative predictive value
